# Comparative transcriptome analysis of the hippocampus from sleep-deprived and Alzheimer’s disease mice

**DOI:** 10.1590/1678-4685-GMB-2019-0052

**Published:** 2020-04-22

**Authors:** Yi Wei

**Affiliations:** 1 Nanjing Forest Police College, Nanjing 210023, China.

**Keywords:** Sleep deprivation, Alzheimer’s disease, hippocampus, Acetyl-CoA, lipid

## Abstract

We did a comparative analysis of the gene expression profiles of the hippocampus from sleep deprivation and Alzheimer’s disease (AD) mice. Differentially expressed genes (DEGs) were identified by comparing the transcriptome profiles of the hippocampus of sleep deprivation or AD mouse models to matched controls. The common DEGs between sleep deprivation and AD were identified by the overlapping analysis, followed by Gene Ontology (GO) and Kyoto Encyclopedia of Genes and Genomes (KEGG) analyses. The results showed that a total of 16 common DEGs showed similar change patterns in both sleep deprivation mice and AD mice. *Sgk1*, *Ly6a*, *Atp6v0e*, *Hspb8*, *Htra1*, *Pdk4*, *Pfkfb3*, *Golm1*, and *Plin3* were up-regulated in the two disorders, whereas, *Marcksl1*, *Fgd1*, *Scarb1*, *Mvd*, *Klhl13*, *Elovl2*, and *Vps29* were down-regulated. Acetyl-CoA metabolic process and lipid biosynthetic process were significantly enriched by those DEGs. The highly expressed DEGs and the two GO terms were associated with neuropathological changes according to the previous studies. As expected, sleep deprivation may contribute the AD development through these common DEGs.

## Introduction

Alzheimer’s disease (AD), a common age-related progressive neurodegenerative disease, is characterized by progressive neuronal loss in the hippocampus and cortex, with the accumulation in the brain of extracellular neuritic plaques caused by β-amyloid (Aβ) peptides as well as intracellular neurofibrillary tangles induced by hyperphosphorylated tau proteins, resulting in irreversible memory loss and declined cognitive functioning (Chen *et al.*, 2017; Area-Gomez *et al.*, 2018). Clinical studies report that up to 45% of patients suffer from sleep disturbances as well as sleep-wake rhythm disturbances, which tends to be one of the earliest symptoms in the development of AD (Peter-Derex *et al.*, 2015). Recently, human and transgenic animal studies show that amyloid deposition and tau aggregation directly cause sleep impairment (Roh *et al.*, 2012; Mander *et al.*, 2015). Besides, dysfunction of neurotransmitter systems responsible for sleep including the cholinergic system, and physiological alterations in the suprachiasmatic nucleus (SCN) and pineal gland, as well as reduction of precuneus volume have been reported to be associated with sleep disturbance in patients with AD (Sarter and Bruno, 1997; Wu and Swaab, 2007; Matsuoka *et al.*, 2018). Therefore, sleep deprivation is initially considered to be a biomarker of a subclinical neurodegenerative process, such as AD.

Recently, many studies demonstrate a relationship between sleep deprivation and AD that sleep deprivation is not only a simple biomarker of AD but a direct contributor to its pathogenesis (Di Meco *et al.*, 2014). Patients with mild cognitive impairment, the early stage of AD, have sleep disturbance before any cognitive impairment, suggesting sleep disturbance may precede clinical diagnosis of AD years in advance (Hita-Yanez *et al.*, 2012; Westerberg *et al.*, 2012). The previous study concludes that sleep deprivation has high comorbidity with many neurodegenerative disorders (Vecsey *et al.*, 2012). Clinical studies show that sleep deprivation increases cerebral Aβ production, and in AβPPswe/PS1∆E9 transgenic mouse model of AD, extended wakefulness results in more Aβ plaques deposition (Kang *et al.*, 2009; Slats *et al.*, 2013). Also, sleep deprivation is correlated with Glycogen synthase kinase 3 (*GSK3*) activation, which causes the phosphorylation of tau and the formation of neurofibrillary tangles (Benedetti *et al.*, 2004). In A βPPswe/PS1∆E9 transgenic mice, chronic sleep deprivation induces an increased number of p-tau (T231) positive neurons and p-tau (T231) protein level, and these pathological alterations still remain even three months after sleep deprivation termination (Qiu *et al.*, 2016). Furthermore, sleep deprivation also contributes to a striking neuronal mitochondrial damage, caspase cascade activation, and neuronal apoptosis in the hippocampus which are all associated with the pathogenesis of AD (Qiu *et al.*, 2016; Area-Gomez *et al.*, 2018). These similar pathological alterations suggest sleep deprivation may be a risk factor for AD development.

The hippocampus plays a crucial role in the formation of spatial, contextual, and declarative memories (Morris *et al.*, 2003), and it is one of the first regions in the brain to suffer damage during AD progression (Braak and Braak, 1991; Braak *et al.*, 1993; Greene and Killiany, 2012). A recent study reports sleep deprivation disrupts hippocampal function and synaptic plasticity, a neural correlate of memory (Prince *et al.*, 2014). It is well-known that sleep deprivation impairs memory, and hippocampus-dependent memory consolidation is particularly sensitive to sleep loss (Porter *et al.*, 2012). The consolidation of memories includes synaptic consolidation and systems consolidation, both of which can be regulated by sleep deprivation (Havekes and Abel, 2017). Thus, the hippocampus may be an important region on memory impairment caused by both sleep deprivation and AD, and the similarities and differences in molecular impacts of the two disorders on hippocampal function should be deserving of attention.

Recent findings have identified several signaling pathways and molecules affected by sleep deprivation or AD in the hippocampus (Polito *et al.*, 2014). However, the related mechanism remains unclear. Here, we compared the gene expression profiles of the hippocampus of sleep deprivation or AD mouse models to its matched controls and identified the conserved differentially expressed genes (DEGs) between them. These common DEGs may be the potential molecular targets involved in the underlying mechanisms.

## Materials and Methods

### Datasets information

Two Gene Expression Omnibus (GEO) datasets were downloaded. GSE53480 dataset included gene expression data of hippocampus from rTg4510 Tau transgenic mice and the littermate wild-type mice. Both of the AD mice models and control mice were C57BL/6J strains (Polito *et al.*, 2014). Mice were euthanized at 4 months of age. GSE33302 dataset included gene expression data of hippocampus from sleep deprivation mice and time-matched non-sleep-deprived control mice. C57BL/6J mice (2-4 months of age) were housed individually on a 12 h/ 12 h light-dark schedule with lights on at 7 am which is set as Zeitgeber time (ZT) 0 (Vecsey *et al.*, 2012). Each mouse was handled daily for 3-6 days before sleep deprivation. Sleep deprivation began between ZT4 and 6 and carried out in the mice’s home cages for5h by gentle handling. Hippocampal dissections were performed immediately following the behavioral treatment.

### Identification of DEGs

All data were extracted and downloaded from Series Matrix File(s). R software was used to pre-process the data via background correction and quantile normalization. “Impute” package (Hastie *et al.*, 2011), a package of R, was applied to complement the missing expression by using the adjacent value. Then, we obtained a file containing available Entrez Gene identifiers and their corresponding expression values.

Limma package, a package of R, was used to identify DEGs in AD mice or sleep deprivation mice compared with their control mice by using empirical Bayes (eBayes) method (Smyth, 2005). The log2 (fold change) of each gene was calculated. To correct for multiple testing, the `fdr’ function was used to adjust the p-value of each gene by using the Benjamini and Hochberg’s approach to control the false discovery rate. Log2(fold change) >0.15 and p <0.05 were set as the threshold. We used sva R package to decrease the Batch effect of GSE53480 and GSE33302 in the previous Differentially Expressed Genes (DEGs) Analysis.

To identify the common DEGs changed in both AD mice and sleep deprivation mice, we overlapped the whole DEGs in the two disorders. And common *DEG*s showing similar change patterns in the two disorders were further sorted out.

### Functional enrichment analysis

The common DEGs showing similar change patterns in the two disorders were used to analyzing functional enrichment analysis by using GO.db (Harris *et al.*, 2004), KEGG.db (Organizer and Goode, 2008) and KEGG REST, R packages. The threshold for significantly enriched GO biological processes and KEGG pathways was set as p-value <0.05.

## Results

### Identification of DEGs in Alzheimer’s disease (AD)

**Figure 1:**
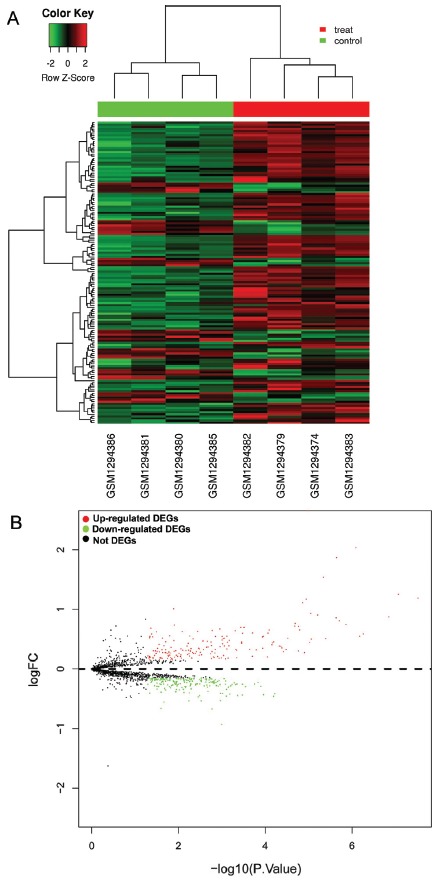
The DEGs in AD mice compared with wild-type mice. A: Heat map analysis. B: Volcano plot analysis. Green represents down-regulated.DEGs, red represents up-regulated DEGs.

GSE53480 dataset including gene expression data of hippocampus from the rTg4510 Tau transgenic mouse models (AD mouse models) and wild-type mice was downloaded and analyzed. Compared to wild-type mice, 382 DEGs were identified in rTg4510 mice hippocampus, at a threshold of p value <0.05 and log2(fold change) >0.15, including 180 up-regulated DEGs and 202 down-regulated DEGs (top 50 up-regulated *DEG*s were shown in Table 1). The cluster analysis showed the expression level of each gene in each sample. In the heat map, the left four columns represent samples from wild-type mice, and the right four columns represent samples from rTg4510 mice) (Figure 1A). The volcano plot revealed the p-value and log2 (fold change) of each gene in rTg4510 mice compared with wild-type mice, and the green and red points represent DEGs (Figure 1B).

**Table 1:** The top 50 up-regulated DEGs in AD mice compared with wild-type mice.

DEGs	Log FC	p-value
Ccl6	2.654661	1.01E-05
C1qb	2.037188	8.02E-07
C1qa	1.869896	2.23E-06
Cd14	1.536667	4.49E-06
Ctsc	1.254297	8.38E-08
Man2b1	1.190078	2.97E-08
Irf8	1.171094	1.11E-05
Cd9	1.110365	1.34E-05
Serping1	1.010443	0.012721
Lcp1	0.933281	8.77E-06
Srgn	0.904922	6.09E-06
Npc2	0.871771	1.40E-07
Itgb5	0.858255	2.27E-06
Serpinf1	0.806563	1.91E-06
Clic1	0.765182	2.00E-05
Tnfrsf1a	0.747682	1.35E-06
Rnase1	0.735547	0.012204
Nfe2l2	0.718568	2.04E-05
Sparc	0.713255	1.68E-05
Lamp2	0.699453	0.000296
Sgk1	0.697813	0.000358
Vsir	0.694245	0.00031
Bgn	0.683047	0.042412
Kcne1l	0.669245	0.001091
Pmp22	0.655938	1.58E-05
Rgs10	0.65401	8.01E-05
Slc22a4	0.644583	0.001629
Aldh1a1	0.641849	0.000484
Hist1h1c	0.631302	0.003537
Ctla2b	0.625781	0.007986
Ifitm2	0.622005	0.031678
Tspo	0.603073	0.000192
Prnp	0.601172	8.51E-05
Gsn	0.598828	0.000327
Sdc4	0.589219	5.40E-07
Cp	0.588932	0.040956
Magel2	0.555638	0.015686
Cpq	0.552448	0.005398
Hspb8	0.549844	0.000749
Creg1	0.548932	6.59E-07
Apoc1	0.547526	0.000109
Lox	0.546016	0.000962
Apod	0.546016	0.044451
Lsp1	0.545156	0.001374
Emp1	0.538203	0.038335
Gm5637	0.535807	0.000113
Cyp1b1	0.522734	0.045007
Serpinf2	0.51612	0.023
Tubb6	0.51375	2.96E-05
Id3	0.513594	0.000866

Among these DEGs, Complement C1q Subcomponent Subunit B (*C1qb*) and Cluster of differentiation 14 (*Cd14*) were highly expressed in AD mouse models compared with wild-type mice. A previous study also showed an increased level of *C1qb* in the senescence-accelerated mouse/prone 8 (*SAMP8*), a suitable animal model to investigate the fundamental mechanisms of age-related learning and memory deficits, and after treated with Huang-Lian-Jie-Du decoction which has the ability to ameliorate the learning and memory function of central nervous system, *C1qb* was decreased (Lu *et al.*, 1998), suggesting that overexpression of *C1qb* may be closely associated with memory impairment. *CD14* is a critical regulator of the microglial inflammatory response modu lating Aβ deposition, and deletion of *CD14* attenuates AD pathology, suggesting its overexpression is also a risk factor for AD (Bi *et al.*, 2017).

### Determination of differentially expressed genes in sleep deprivation

**Figure 2:**
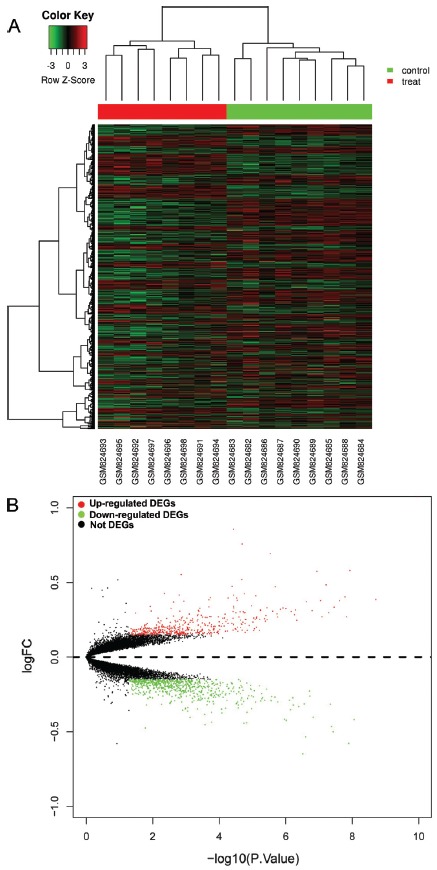
The DEGs in sleep deprivation mice compared with non-sleepdeprived control mice. A: Heat map analysis. B: Volcano plot analysis. Green represents down-regulated. DEGs, red represents up-regulated DEGs.

GSE33302 dataset, including gene expression data of hippocampus from sleep-deprived mice and time-matched non-sleep-deprived control mice, was analyzed. Compared to control mice, a total of 1149 genes were differentially expressed in sleep deprivation mice at a threshold of p-value <0.05 and log2(fold change) >0.15, which consisted of 470 up-regulated genes and 679 down-regulated genes (top 50 up-regulated DEGs were shown in Table 2). A heat map showing the expression levels of all 1149 DEGs were generated. The left eight columns represent samples from sleep deprivation mice, and the right nine columns represent samples from non-sleep-deprived control mice (Figure 2A). The volcano plot revealed the p-value and log2(fold change) of each gene in sleep deprivation mice compared with nonsleep-deprived control mice, and the green and red points represent differentially expressed genes (Figure 2B).

**Table 2:** The top 50 up-regulated DEGs in sleep deprivation mice compared with non-sleep-deprived control mice.

DEGs	Log FC	P Value
C330006P03Rik	1.075404	1.04E-10
Gm19439	0.856981	3.64E-05
Fos	0.757198	1.99E-05
Arc	0.692398	2.87E-06
Zmym1	0.580331	1.15E-08
Fam46a	0.575429	1.13E-07
Sgk1	0.553333	0.001363
Klf2	0.519179	2.03E-05
Hist2h3b	0.484559	5.94E-08
Hspb1	0.474444	4.00E-05
Pglyrp1	0.470768	2.00E-06
Sult1a1	0.426495	0.004354
Manf	0.425482	1.72E-05
Slc39a2	0.415629	0.001572
Ppp1r3g	0.41489	3.61E-05
Thbs4	0.410694	7.70E-06
Nostrin	0.403824	1.61E-08
Hist1h3d	0.398284	0.00018
Gjb6	0.395809	2.93E-05
Plekhf1	0.395282	5.27E-05
Prkab2	0.385805	1.89E-09
Alox12b	0.384485	0.000199
Nr4a1	0.381311	2.83E-05
Hspa5	0.379371	8.82E-08
P4ha1	0.379167	2.35E-08
1500032P08Rik	0.374252	0.001125
Creld2	0.371924	2.34E-07
Gm3515	0.368799	0.002315
Tsc22d3	0.344395	1.04E-05
Nfil3	0.339857	3.60E-05
Plin4	0.339444	0.011519
Ddit4	0.336401	0.000199
Hist1h3a	0.334779	3.41E-08
Rasl10a	0.330384	3.56E-06
Klf4	0.329763	3.55E-05
Hrk	0.329359	1.18E-06
Mfsd2a	0.327831	0.000374
Hbb-bt	0.326426	0.002756
Htr1a	0.325029	9.61E-07
Gpt2	0.320188	1.42E-06
Med20	0.315592	4.95E-07
Dnajc28	0.315429	5.62E-06
Nfkbia	0.312606	0.000415
Gm5553	0.311924	5.58E-06
Hdac4	0.310993	1.57E-07
8430408G22Rik	0.309265	0.010796
A330023F24Rik	0.30857	0.00149
Smim3	0.308505	0.000401
St5	0.307945	2.41E-06
Dio2	0.306009	6.69E-07

Among these DEGs, Fos transcription factor family (*Fos*) and activity-regulated cytoskeleton-associated protein (*Arc*) were highly expressed in sleep deprivation mice compared with non-sleep-deprived control mice. The overexpression of *Fos* in hippocampal neurons in AD has also been reported by Lu *et al.*, 1998. *Arc* is a key factor for AD, plays a crucial role in synaptic plasticity, learning, memory, and Aβ generation, and the *Arc* gene in human confers susceptibility to AD in Han Chinese (Bi *et al.*, 2017).

### Potential molecular targets involved in the impact of sleep deprivation on AD pathogenesis

Here we overlapped the DEGs between sleep deprivation mice and AD mice. 38 common DEGs were identified, and 16 of them showed a similar change (Table 3). The increased DEGs included serum/glucocorticoid regulated kinase 1 (*Sgk1*), lymphocyte antigen 6 complex (*Ly6a*), V-type proton ATPase subunit e 1 (*Atp6v0e*), heat shock protein family B member 8 (*Hspb8*), high-temperature requirement serine peptidase A1 (*Htra1*), pyruvate dehydrogenase kinase 4 (*Pdk4*), 6-phosphofructo-2-kinase/fructose-2, 6-biphosphatase (*Pfkfb3*), Golgi membrane protein 1 (*Golm1*) and perilipin-3 (*Plin3*) were up-regulated in the two disorders. Meanwhile, the rest of them were decreased, including Myristoylated Alanine-Rich C-kinase Substrate protein 1 (*Marcksl1*), guanine-nucleotide exchange factor (*Fgd1*), sucrose-6-phosphate dehydrogenase (*Scarb1*), Mevalonate Diphosphate Decarboxylase (*Mvd*), Kelch-like family member 13 (*Klhl13*), ELOVL fatty acid elongase 2 (*Elovl2*) and Vacuolar protein sorting-associated protein 20 (*Vps29*). Among them, *Sgk1* was highly expressed with the highest fold changes in both sleep deprivation and AD mice.

**Table 3:** A total of 16 common DEGs showed similar change patterns in both sleep deprivation mice and AD mice.

Common DEGs	Log FC in sleep deprivation mice compared with nonsleep-deprived control mice	Log FC in AD mice compared with wild-type mice
Sgk1	0.553333333	0.6978125
Ly6a	0.278660131	0.510390625
Atp6v0e	0.251478758	0.39078125
Hspb8	0.161646242	0.54984375
Marcksl1	-0.160261438	-0.534609375
Htra1	0.209203431	0.406328125
Pdk4	0.213876634	0.346744792
Fgd1	-0.168006536	-0.422604167
Pfkfb3	0.220535131	0.290546875
Scarb1	-0.182712418	-0.341432292
Golm1	0.249387255	0.194140625
Plin3	0.160951797	0.291510417
Mvd	-0.162626634	-0.258515625
Klhl13	-0.172769608	-0.238541667
Elovl2	-0.201650327	-0.193359375
Vps29	-0.161968954	-0.1528125

**Figure 3:**
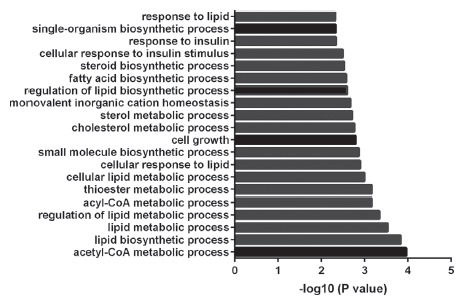
Top 20 significantly enriched GO biological processes.

To understand the associated pathways of these common genes, GO and KEGG were conducted on these 16 genes. This showed that the common DEGs were mainly related to acetyl-CoA metabolic process and lipid biosynthetic process. Acetyl-CoA metabolic process included *Pdk4* and *Mvd*, whereas lipid biosynthetic process consisted of *Scarb1*, *Pdk4*, *Elovl2*, and *Mvd*. The top 20 significantly enriched GO biological processes were shown in a histogram (Figure 3). KEGG pathway analysis showed that these DEGs were mainly enriched in Phagosome.

## Discussion

Here we performed acomparative analysis of the transcriptome profiles of mouse hippocampus of sleep deprivation or AD and identified the common DEGs between them, which were highly related to acetyl-CoA metabolic process and lipid biosynthetic process. These common DEGs may explain the potential mechanism of how sleep deprivation affecting AD development.

Although either sleep deprivation or AD can cause hippocampus dysfunction, the related molecular changes of the hippocampus in mouse models of the two disorders were different. The number of DEGs in sleep deprivation mice (1149 genes) is nearly three times more than that of AD mice (382 genes). Overlapping of the DEGs between them identified 16 common DEGs with the same change direction. Among these common DEGs, the previous studies suggest that some of the induced genes, such as *Sgk1* or *Htra1* or *Pfkfb3*, may play a protective role in AD development while some of them (e.g. *Golm1*) may aid in AD development. In detail, *Sgk1* has been reported to be correlated with the inhibition of Aβ deposition and the improvement of cognitive function and to play an important role in neuronal plasticity, spatial memory and fear-conditioning memory in the hippocampus (Izumi *et al.*, 2018). Htra1 is demonstrated to cleave Tau (Poepsel *et al.*, 2015) while Tau was proven to be the trigger and bullet in Alzheimer disease pathogenesis (Poepsel *et al.*, 2015). Meanwhile, Pfkfb3 is the rate-limiting enzyme for glycolysis, and inhibition of Pfkfb3 has been proved to cause an accumulation of amyloid protein and vulnerability to Aβ cytotoxicity (Fu *et al.*, 2015). Therefore, these three genes were induced by sleep deprivation or AD probably due to a feedback mechanism. In contrast, the genetic variation of *Golm1* was found to be associated with AD. For example, a polymorphism of *Golm1*, rs10868366, has been identified as an AD risk factor (Inkster *et al.*, 2012). Taken together, some of the common DEGs have been reported to be involved in AD development, suggesting an investigation of the role these overlapped genes may aid to understand how sleep deprivation may promote AD development.

Our further GO biological processes analysis showed that the 16 common DEGs were mainly enriched in the acetyl-CoA metabolic process and lipid biosynthetic process. Pyruvate-derived acetyl-CoA is a principal direct precursor substrate for bulk energy synthesis in the brain (Bielarczyk *et al.*, 2015). A β-induced deficits in acetyl-CoA are confined to mitochondrial and cytoplasmic compartments of Tg2576 mice nerve terminals, which is the early primary signal for neurodegeneration (Bielarczyk *et al.*, 2015). These findings suggest a critical role of acetyl-CoA metabolic process in AD. It is reported that lipid metabolism is significantly influenced in neurodegenerative diseases, such as AD, and the dysfunctions can further cause abnormal levels of certain lipids in the brain, cerebrospinal fluid and plasma (Zarrouk *et al.*, 2018). Previous studies have reported several lipid biomarkers for AD, including cholesterol, oxysterols, fatty acids, and phospholipids, some of which have a prognostic and diagnosis value, suggesting lipids play a role in the pathogenesis of AD (Zarrouk *et al.*, 2018).

There are some limitations to our current manuscript. First, our analyses were done on mouse samples, instead of human samples. However, tissue from sleep-deprived humans is to be difficult to acquire. Therefore, we further analyzed the online databases about human brain tissues with AD. DEGs from human Alzheimer’s disease brains (GSE36980 and GSE5281 datasets) were identified using the same cutoff (logFC >0.15, p <0.05) as our current analyses, followed by overlapping with the 16 common DEGs in sleep-deprivation mice and AD mice (Table 3). Three (*HSPB8*, *FGD1*, *GOLM1*) and five (*MARCKSL1*, *PFKFB3*, *PLIN3*, *MVD*, *VPS29*) overlapped genes were found in GSE36980 and GSE5281 datasets, respectively. It suggests that these genes may play a vital role in the development of human Alzheimer’s disease. Second, the batch effect may exist. Although the two datasets used in our study were done on C57BL/6J mouse strains, they are finished in a different lab in different conditions so that we cannot exclude the batch differences may exist in these two independent experiments. Last, although the investigation of the role of these common genes in AD can aid in the understanding of AD development, we should remember that not all sleep-deprived individuals will develop AD. Other factors, such as genetic background, other living habits, and general health conditions, should be considered even though the individuals may have similar changes of these 16 DEGs.

In conclusion, taking advantage of the online databases, we systematically analyzed the transcriptome profiles of mouse hippocampus of sleep deprivation and AD, followed by the identification of the common DEGs between them. Most of the common genes, highly enriched in acetylCoA metabolic and lipid biosynthetic processes, are reported to be associated with neuropathological changes. These findings suggest that sleep deprivation causing neuropathological changes in mouse brains may contribute the AD development by dysregulation of these common DEGs.
